# The Application of Electrometallurgy in Dental Practice

**Published:** 1901-08

**Authors:** Wm. Leon Ellerbeck

**Affiliations:** Philadelphia


### International Dental Journal.

Vol. XXII. August, 1901. No. 8.

### Original Communications.[1]

### THE APPLICATION OF ELECTROMETALLURGY IN DENTAL PRACTICE.

BY WM. LEON ELLERBECK, PHILADELPHIA.

Of the several metal base-plates which have been employed for artificial dentures, the electrodeposit, so-called galvanoplastic base-plate, is the only one which gives us absolute adaptation to the model. Of this one may well judge when it is considered that a thumb- or finger-mark accidentally left on the carbonized surface of a plaster model prepared for the deposit may be clearly defined on the approximating surface of the metal after the deposition has taken place.

While the electrodeposit silver base, as made by many, has proved unsuccessful to a degree which would practically prohibit its recommendation over plates made of other materials and by other methods, still in the hands of some skilled in the art of electrometallurgy, light, durable, and generally satisfactory plates have been produced. It is but natural, then, that the query arise as to whether the method or the man is most at fault, in the general discontinuance of this base, which, while applicable to all

[1] The editor and publishers are not responsible for the views of authors of papers published in this department, nor for any claim to novelty, or otherwise, that may be made by them. No papers will be received for this department that have appeared in any other journal published in the country.

cases where the mere question of supplying artificial teeth is concerned, is strongly indicated in at least two classes of mouths which may present.

First, where the roof of the mouth is broad and flat and excessive alveolar absorption has taken place. Here the utmost possibilities for securing the necessary perfect adhesion to insure retention of the denture are obtainable in the electrodeposit base.

Secondly, in mouths with a high contracted arch and deeply undercut alveolar ridges—such a case as would involve great difficulty in the swaging process—the deposit plate may be made with comparative ease, the only difficulties being those met with in the proper management of the electric current and the plating solution. The chief objections to the electrodeposit silver base are the occasional weakness due to porosity of the metal where the plating has been unskilfully performed,—and in addition its tendency to discolor through the action of the sulphides of the mouth,—and because of the difficulty in keeping the sulphur of the rubber used for attachment from combining chemically with the silver.

While it is easily possible for most practitioners to allot a small part of their laboratory for the necessary batteries and plating solutions and give the time and attention necessary for successful results, still, just because of this necessity for very careful observation of detail, it is hardly probable that the busy practitioner will ever take up this method and make it a feature of his general work. There is, however, nothing impracticable in its employment by the prosthetic dentist who makes a specialty of working for the profession; on the contrary, the fact that a great many plates may be made at one time should appeal to him, especially if the objections cited could be largely or wholly overcome. With a view of stimulating further experimentation by those experimentally inclined, I give the general directions to follow in procuring the silver base, and trust that the few suggestions which I make may be of value in leading to the perfection of this method, which I feel has not been given the scientific attention which it seems to merit and which if perfected would be of considerable importance in dental prosthesis.

Having obtained in the usual manner an accurate impression of the mouth to be fitted, a model is run and trimmed as if a vulcanite plate were to be made. The air-chamber may be made of thin sheet lead or tin and adjusted to fit tightly against the plaster, or it may be made by cutting a shallow chamber in the impression before running the model. The metal chamber is, however, preferable, on account of its having better conducting properties than the carbon.

After having placed the air-chamber in the proper position, a fine groove should be made on the model to show the limiting line or margin of the plate. If any uncertainty exists as to where it should be allowed to extend, a base plate of wax may be made, tried in the mouth, and trimmed until satisfactory, then placed on the model and a line made on the plaster to correspond exactly with the margin of the wax. After this the groove should be made, using the line as an indicator. This line, of course, appears on the plate as a faint ridge, and serves as a guide in trimming the metal to the proper place. A wire, preferably of silver, is passed through the plaster at a place a little distant from where the posterior margin of the plate is to be. It should be bent over the edge of the model and twisted around the main portion of the wire until it is slightly embedded in the plaster.

The model should now be placed in a pot of boiling beeswax until it is thoroughly saturated. A few trials will enable one to tell when it has been soaked enough. The wax penetrates throughout the plaster, making it hard or horny and impervious to moisture, thus preventing it from disintegrating when placed in the depositing solution. It also renders the surface suitable for the reception of the graphite or whatever is used for the conducting coat. After the model has cooled the surface is carbonized with finely powdered graphite, the Ceylon variety being preferable, since it contains a large proportion of carbon and very little silicon and other impurities which would interfere with its conducting properties. The carbon is applied with a camel's-hair brush having a thick body of short hairs. Breathing on the model occasionally facilitates the adhesion. When the surface is perfectly black and bright the excess powder should be blown off, and that which adheres beyond about a sixteenth of an inch from the marginal line should be scraped away. It is necessary to leave this slight excess because there is a tendency for the deposited metal to become granular or nodular along the edges, and the excess admits of a little trimming, so that when the metal is filled to the marginal mark the deposit will be hard and reguline.

To remove any oil or grease that may have gotten on the surface, it is well to pour over the model a dilute solution of potassium hydrate, and then wash well with water. The model thus prepared is ready for the reception of the deposit. There are several solutions which may be used for this purpose, but the best and the one most frequently employed is a solution of argento-potassic cyanide made by dissolving argentic cyanide in potassium cyanide until the right amount of silver is in solution. The reaction which takes place is shown in the following simple equation:

$$AgCN + KCN = AgCN, KCN$$

Some free potassium cyanide is added for the purpose of keeping the solution of the desired strength. The carbonized model is used as the cathode or receiving surface, and is consequently attached by the conducting wire to the negative pole of the battery, or whatever is used to supply the current. To the positive pole is attached an anode of pure silver plate, the amount of surface immersed in the solution depending upon the strength of the current necessary to produce a good deposit, and upon the area of the cathodal or receiving surface. When the current is applied silver is deposited, beginning at the junction of the feed-wire and the carbon of the model and spreading gradually over the surface, presenting a beautiful frosty white appearance. The action is allowed to continue until the plate reaches the proper thickness, a slight excess being allowed for dressing. It should then be taken from the solution and thoroughly washed with water to remove any traces of the poisonous potassium cyanide. Here I would suggest that a possible way to overcome any weakness due to porosity would be to subject the plate while still closely adherent to the model to the shot-swaging process, which I believe will make the metal denser and more rigid. The wax-soaked model being less fragile than the unwaxed cast, it is less liable to spread or crack. After swaging, the plate should be removed from the model. To accomplish this first apply a gentle heat until the wax begins to bubble from the plaster, then place a knife-blade under the edge of the metal. The plate can be pried off without difficulty. It should then be trimmed with a file to the marginal line, which corresponds to the depression made in the model. The rough nodular portions should be ground off with a corundum wheel, and the surface of the plate polished to a high degree, using felt wheels and pumice, scratch-brushes, buff-wheels and rouge, etc. The brighter and better the polish of the silver the greater are the chances for a good gold deposit. After this the plate is placed on a model for support, and is speared with a pointed chisel or gouge, slight elevations being made along the ridge and wherever the rubber is to be attached. It is then ready for the gold bath, in which it should be placed and heavily gilded to prevent chemical action between the silver and the sulphur of the rubber. This done, it is ready for the teeth. From this point proceed in the same way as when making a rubber attachment to a gold plate. After vulcanizing, the piece should be again polished. In this operation some of the gold deposit is removed, and must be replaced to prevent the plate from tarnishing. It is therefore again placed in the gold bath until it receives a good thick deposit. It should be thoroughly washed to remove any traces of potassium cyanide, and then scratch-brushed and highly polished, after which it is ready for the patient.

DEPOSITING SOLUTIONS.

From two to four ounces of silver per gallon are sufficient for silver-coating metallic surfaces, but in order to get the first thin film to deposit on the carbon, a heavier solution is desirable, as it is difficult to start the plating with a weak solution. Ten ounces to the gallon makes a good solution for the purpose, but once the film is made it is transferred to the weak solution, and while more time is required in order to obtain the desired thickness, it is more uniform and tough when once obtained. Having decided upon the amount we wish to make, the process is a simple chemical calculation. If, for example, we wish to make one gallon of the strong solution, ten ounces of silver should be converted into argentic nitrate in the usual manner, dissolved in water, and the silver precipitated out as a cyanide by the addition of potassium cyanide, care being taken not to add any more than just sufficient to precipitate all the metal, as the silver salt is soluble in an excess of this agent. The reaction is as follows:

$$AgNO_3 + KCN = AgCN + KNO_3$$

The precipitate is collected on a filter-paper and washed with distilled water to remove any traces of potassium nitrate. It is then dissolved in potassium cyanide, the amount necessary to affect the solution being easily calculated in the following manner:

First determine by simple proportion how much silver cyanide was produced from the silver employed,—107.6 (once the atomic weight of silver) : 133.6 (molecular weight of silver cyanide) :: ten ounces of silver (amount used) : $x$ (weight of silver cyanide produced). Then 133.6 (once the molecular weight of silver cyanide) : 65 (once the molecular weight of potassium cyanide) :: $x$ (amount of silver cyanide) : $x$ 1 (total amount of potassium cyanide necessary to combine with silver cyanide).

The potassic salt is dissolved in distilled water, and to it is added one-half more for free cyanide, the necessity for which will be presently explained. After the silver cyanide is dissolved the solution should be made up to one gallon. It is then ready for use.

Another way to make the solutions where facilities for chemical work are not handy is to calculate the amount of potassium cyanide necessary to make the argento-potassic salt, and add one-half more for free cyanide. Then take a large silver anode and a much smaller silver cathode, the weights of which are known, attach them to the poles of a battery, immerse in the solution, and weigh from time to time. The current should be used as strong as possible without throwing the deposit down as a powder. The silver is dissolved from the anode more rapidly than it is deposited on the cathode. As a consequence the solution grows constantly stronger. This action should be kept up until the difference in weight of the loss of the anode and the increase in weight of the cathode shows the solution to be of the desired strength. The reactions which take place are as follows:

$$KCN + Ag = AgCN + K$$

$$K + H_2O = KOH + H$$

$$AgCN + KCN = AgCN, KCN$$

In many instances where there are deep depressions in the model which is to be coated it is difficult to make the deposit spread in these parts. Where such conditions exist, extra guides of fine silver wire should be attached to the main feed and bent so that their ends will rest at the bottom of the depression. This will likely overcome the difficulty.

Where good graphite cannot be obtained, its conducting power may be increased by gilding it. To do this, dissolve one part of gold chloride in one hundred parts of sulphuric ether in a deep glass vessel, add fifty parts of the powdered graphite, mix them thoroughly, and expose the mixture to the sunlight, stirring it at different intervals until it is dry; it can then be applied in the same manner as the graphite. Where for any reason the silver cannot be made to start directly on the carbon, a thin trace of copper may be deposited, after which the silvering may be readily accomplished, the copper being easily removed by dilute nitric acid after the plate is removed from the model, the gold used in plating filling up the place of the removed copper, though the very fine lines may not be reproduced. A good copper solution may be made by dissolving eight ounces of pure copper sulphate in one part of boiling water, adding one of pure sulphuric acid, and diluting to one quart.

### MANAGEMENT OF PLATING SOLUTIONS AND CONTROL OF THE ELECTRICAL CURRENT EMPLOYED IN THE DEPOSITION.

As before mentioned, a good plating solution should contain one equivalent (sixty-five parts) of pure potassium cyanide, one equivalent (133.6 parts) of argentic cyanide, and from thirty to fifty per cent. of free cyanide, with sufficient water to make a thin liquid. It should be remembered that ordinary commercial potassium cyanide is very impure, and the absolute amount of potassium cyanide present varies considerably. This should be taken into account in making depositing solutions, otherwise the proportion will not be at all accurate. If possible the chemically pure cyanide should be employed, because the commercial article contains impurities such as potassium carbonate and potassium chloride, which salts are likely to interfere with the perfect working of the solutions. The German cyanide is to be preferred.

The necessity for having free cyanide arises from the fact that in the working of the solution there is formed at the anode insoluble silver cyanide, and free potassium cyanide is necessary to combine with it and form the soluble double salt. Simultaneously with the formation of this compound at the anode, potassium cyanide and free cyanogen are liberated at the cathode, due to the deposition of the silver. It requires some time for those substances to mix with the liquid and reach the dissolving plate, therefore the extra potassium cyanide is added to allow the deposit to proceed uninterruptedly. It is necessary to have sufficient water to form a thin liquid because the double salt of silver and potassium, being specifically heavier than the solution, tends to sink to the bottom. On the other hand, the cyanogen and potassium cyanide set free at the cathode, being specifically lighter, tend to rise to the top; at the same time, due to capillarity, these substances tend to diffuse into the surrounding liquid, and the thinner the solution the more mobile are its particles and the more rapidly the diffusion takes place. To prevent the fluid from settling into strata of different densities, it should occasionally be stirred.

There have been several theories advanced to explain the phenomena of electrolysis. One which appears plausible is the polarization of molecules, which is that when the salt is decomposed and the metal liberated, the substance with which it was in combination, being in a nascent state, expresses a stronger affinity for the metal of the molecule next to it than the substance with which it is already combined, and thus an interchange takes place repeated through a direct chain of molecules, the liberated substance of the molecule decomposed exerting a like influence upon the succeeding one until the anode is reached, where, the same nascent condition prevailing, the substance attacks it and becomes satisfied. The solution should be stirred frequently to insure homogeneity of the liquid, also occasionally filtered to remove extraneous particles held in suspension. That there is a necessity for frequent stirring because of a tendency to the formation of different strata of different densities may be easily determined after the solution has been used for some time without being disturbed. Immerse near the surface a piece of metal to which is attached a wire, and immerse in the lower portion another piece of the same metal, then attach the wires to a galvanometer. The needle is deflected. This is brought about by the electrical current produced by the action of one metal in two liquids. It goes to show the difference in composition of different portions of the depositing solution. A practical demonstration of this occurs at a time when the battery for some reason becomes suddenly weak. The silver deposited upon the model will be redissolved and deposited back on the anode. This is brought about by the liquids surrounding the anode having, through long continued use, become saturated with silver and that about the cathode having become full of free cyanide. The two electrodes form a voltaic battery (one metal in two liquids), and send a current opposite in direction to that supplied by the battery used in depositing. The electrical current used in small operations, such as would be required in the dental laboratory, is supplied by voltaic cells such as Smee's or Bunsen's, but for large operations where a considerable amount of work is done, as in electroplating establishments, the magneto-electric machines are employed, they being more uniform in action and necessary to insure uniform results. Much depends upon the control of the current. If the metal is deposited upon the feed-wire as a grayish-brown powder, and does not extend to the model, the blackleading is likely imperfect. If, however, it extends to the model and is still brown and powdery, it shows that the current is too strong. The following simple experiment will show what is necessary to control the current in order to obtain good results:

Connect one pole of the battery to one of the terminals of the galvanometer; to the other terminal attach the wire having the silver plate. See that the galvanometer points to zero, then immerse the silver strips a few inches apart. Observe that the deflection is decreased by increasing the distance between the strips, and increased by bringing them closer together. Observe that the deflection is decreased by lessening the amount of metal immersed; either pole or both. The same results may be obtained by lessening the extent of immersion of the different materials used in the battery solution. Again, substitute for a part of the silver wire a small piece of German silver. Notice that the resistance is greater and that the needle is not deflected to such an extent as with silver alone. Having these few facts in mind, the operator should be able to control the current perfectly.

Generally speaking, the area of the anodal surface should be equal to the area of the cathode, the number of plates being made at one time gauging the size of the silver plate, the strength of the current, of course, modifying this some.

The gold-plating solution may be made in the same manner as the silver bath, of course using the cyanide of gold in place of the silver salt, and where the battery method is employed using a gold cathode in place of silver. Gold solutions should contain about one ounce of the metal to the gallon, and should be used at a temperature ranging from 90° to 130° F., with a current of two cells.

Should the plates at any time become tarnished in the mouth, they may be readily regilded.

